# Investigating the Antioxidant and Acetylcholinesterase Inhibition Activities of *Gossypium herbaceam*

**DOI:** 10.3390/molecules18010951

**Published:** 2013-01-14

**Authors:** Yongxin Zhao, Jun Dou, Tao Wu, Haji Akber Aisa

**Affiliations:** 1 Xinjiang Key Laboratory of Plant Resources and Natural Products Chemistry, Xinjiang Technical Institute of Physics and Chemistry, Chinese Academy of Sciences, Urumqi 830011, China; 2 The University of Chinese Academy of Sciences, Beijing 100039, China

**Keywords:** *Gossypium herbaceam*, antioxidant activity, Alzheimer’s disease, flavonoid

## Abstract

Our previous research showed that standardized extract from the flowers of the *Gossypium herbaceam* labeled GHE had been used in clinical trials for its beneficial effects on brain functions, particularly in connection with age-related dementia and Alzheimer’s disease (AD). The aim of this work was to determine the components of this herb and the individual constituents of GHE. In order to better understand this herb for AD treatment, we investigated the acetylcholinesterase (AChE) inhibition and antioxidant activity of GHE as well as the protective effects to PC12 cells against cytotoxicity induced by tertiary butyl hydroperoxide (tBHP) using *in vitro *assays. The antioxidant activities were assessed by measuring their capabilities for scavenging 1,1-diphenyl-2-picylhydrazyl (DPPH) and 2-2'-azinobis-(3-ethylbenzothiazoline-6-sulfonic acid) (ABTS) free radical as well as in inhibiting lipid peroxidation. Our data showed that GHE exhibited certain activities against AChE and also is an efficient free radical scavenger, which may be helpful in preventing or alleviating patients suffering from AD.

## 1. Introduction

Alzheimer’s disease (AD) is the most prevalent form of dementia, arising as a result of malfunctions of different biochemical pathways. Multiple pathogenic factors including aggregated amyloid-*β*-peptide (A*β)* and *tau* protein, excessive transition metals, oxidative stress and reduced acetylcholine level have been implicated in AD pathology [1].

There have been great interests in the development of new drugs in the field of AD. Neuropathological occurrence of AD symptoms and cognitive deficits were found to be consistent with the presence of Aβ plaques and cholinergic deficiency due to the degeneration or atrophy of cholinergic neurons in the basal forebrain [2]. Thus, cholinesterase inhibition has been one of the mainstays for treatment of AD and is considered to be a promising strategy for dementia therapy. To date most of the drugs approved and licensed for the disease have been AChE inhibitors, such as donepezil and galanthamine [3]. Unfortunately, these drugs can cause undesirable side effects and they are largely ineffective for treating severe AD cases. Therefore, it is compulsory to search for new anti-AD drug candidates. There have been a large number of examples which have repeatedly pointed to the need of expanding the exploration of Nature as a source of bioactive compounds that may serve as the leads or scaffolds for further chemical elaboration [4,5].

Oxidative stress has been considered a mechanism involved in the pathogenesis of AD, and it has also played a major role in the aging process [6,7]. Oxidative damage by free radicals has been well investigated within the context of oxidant/antioxidant balance [8]. Low levels of reactive oxygen species (ROS) and reactive nitrogen species (RNS) are important for maintenance of neuronal function, though elevated levels can lead to neuronal cell death. Antioxidants may scavenge ROS and may consequently attenuate inflammation pathways. Tertiary butyl hydroperoxide (tBHP) is a well established cytotoxin and oxidative stress inducer, and the rat pheochromocytoma cell (PC12) model is well suited for our research purposes. AD is known to be associated with amyloid-β plagues eliciting neuronal oxidative stress. Free radical damage has been identified as an underlying mechanism for specific neurodegenerative diseases. The brain consumes large amounts of oxygen and therefore produces a comparatively large amount of free radical by-products. The increasing interest in the measurement of antioxidant activity of different plant samples is derived from the overwhelming evidence of the importance of ROS [9]. Several methods have been developed to measure the antioxidant activity in biological samples, including to DPPH radical scavenging, ABTS radical scavenging and inhibition of formation of thiobarbituric acid reactive species (TBARS) [10].

*Gossypium herbaceam *extracts (GHE), an active ingredient from *Gossypium herbaceam*, has been used for a long time as an ethical herb by the Uygur people in Xinjiang, China, to treat mental retardation. It is a complex mixture mainly containing flavonols, such as quercetin, isoquercetrin and quercimeritrin. Each constituent of GHE has been identified, and their metabolites have been profiled [11]. We have previously demonstrated that GHE has antioxidant stress properties in a rat model of AD induced by Aβ peptides, and exerted a positive effect on learning and memory impairment in rats induced by intracerebral injection of ibotenic acid [12]. The purpose of this study is to continue to evaluate the antioxidant and AChE inhibition of GHE. All tested compounds were isolated and obtained from GHE, using ^1^H-NMR and ^13^C-NMR analysis [13] as well as comparison of their basic spectral characteristics [11].

## 2. Results and Discussion

### 2.1. High-Performance Liquid Chromatography (HPLC) Analysis of the Crude Sample and Structural Identification

**Figure 1:**
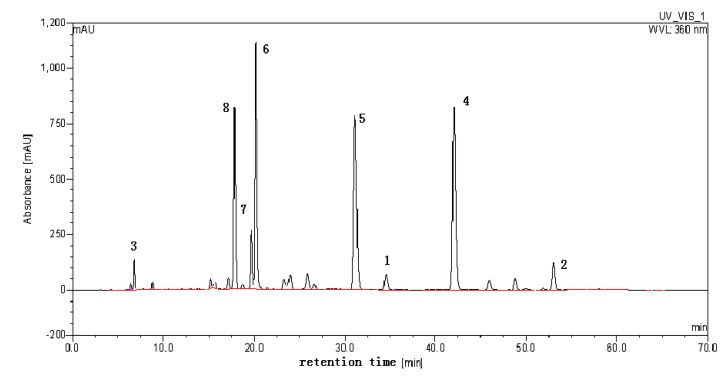
HPLC analysis of the GHE and peak fractions from GHE.

The crude extract of *Gossypium herbaceam* was analyzed using HPLC. The result indicated that it contains several different types of flavonols. HPLC analysis was performed using a Dionex (Sunnyvale, CA, USA) system comprising automated sample injector (ASI-100), pump (P680), thermostatted column compartment (TCC-100), ultra-violet detector (UVD-170U) running Chromeleon 6.80 software. The effluent was monitored by UV detection at 360 nm. Separation column: an inertsil ODS-SP column (4.6 mm I.D. × 250 mm, 5 μm); column temperature: 35 °C; the mobile phase: a linear gradient of acetonitrile (A), methanol (B), and 0.2% formic acid (C) that follows: A-B-C (10:10:80, v/v) to A-B-C (15:15:70, v/v) in 15 min, then to A-B-C (0:55:45, v/v) in 35 min, then to A-B-C (0:80:20, v/v) in 6 min, and finally to A-B-C (0:80:20, v/v) in 8 min. Flow rate: 1.0 mL·min^−1^. Column temperature: 35 °C (Figure 1).

**Figure 2:**
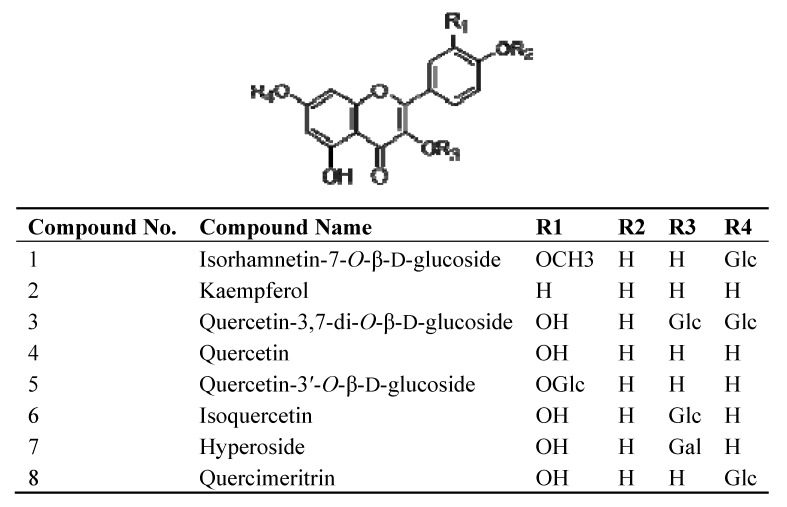
Chemical structures of the compounds from GHE.

The structural identifications of compounds from GHE were carried out using ^1^H-NMR, ^13^C-NMR and high-performance liquid chromatography multistage tandem mass spectrometry (LC-MS^n^) [9]. Peaks 2, 4, 5, 6, 7, 8 were successfully identified as kaempferol, quercetin, quercetin-3'-*O*-β-D-glucoside, isoquercetin, hyperoside and quercimeritin, respectively [13]. Peak 1 was identified as isorhamnetin-7-*O*-β-D-glucoside and Peak 3 was identified as quercetin-3,7-di-*O*-β-D-glucoside according to the ^1^H-NMR, and ^13^C-NMR, and by comparing with our previous report on LC-MS^n^ data [11,14] as well as report from Wang and Huang [15,16]. The molecular structures of the compounds are shown in Figure 2.

### 2.2. Inhibition of Acetylcholinesterase and Antioxidant Activities

The antioxidant activity of compounds from GHE as well as GHE itself were examined by two free radical (DPPH and ABTS) scavenging tests in the well established microplate format. At the same time we also investigated the acetylcholinesterase (AChE) inhibition and lipid peroxidation. The results indicated that the GHE presented a certain degree of inhibition of AChE and antioxidant activities. The IC_50_ value of DPPH and ABTS radical-scavenging activities were found to be 13.28 and 1.12 µg/mL, respectively. IC_50_ values of antioxidant activity and AChE inhibition of GHE are summarized in Table 1.

**Table 1:** GHE (μg/mL) needed to inhibit acetylcholinesterase and antioxidant activities by 50%.

Extracts ^a^	DPPH ^b^	ABTS ^c^	TBARS ^d^	AChE ^e^
GHE	13.28	1.12	3.57	28.09

^a^ Mean values of three replicates are given. Mean ± SD, n = 3; ^b^ Compared to Vit C (IC_50_ = 8.9 μg/mL), IC_50_ for inhibition of DPPH radical formation; ^c^ Compared to Vit C (IC_50_ = 3.1 μg/mL), IC_50_ for inhibition of ABTS radical formation; ^d^ Compared to Vit C (IC_50_ = 5.2 μg/mL), IC_50_ for inhibition of peroxidation of lipids, estimated as thiobarbituric acid reactive substances (TBARS); ^e^ Compared to Huperzine A (IC_50_ = 0.0104 μg/mL), IC_50_ for inhibition of AChE.

In addition to the activities of the crude extracts, the isolated compounds were also studied. Table 2 presents enzyme inhibiting and antioxidant activities for the eight representative compounds isolated from GHE. For those compounds which have more than 50% inhibition in the tested concentration, their IC_50_ values were measured (Table 2) to further evaluate the structure-activity relationship.

The DPPH radical activity test was used to measure the hydrogen atom or electron donor capacity of the samples to the stable radical DPPH formed in liquid solution. Thus, it measures the capacity of the samples to scavenge free radicals in solution [17]. Proton radical scavenging is an important attribute of antioxidants. Although the DPPH radical scavenging abilities of GHE was a little lower than that of Vit C, it was evident that GHE did show the proton-donating ability and could serve as free radical inhibitors or scavengers, acting possibly as primary antioxidants. ABTS, a protonated radical, has characteristic absorbance at 734 nm which decreases with the scavenging of the proton radicals [18]. The ABTS free radical activity of GHE was greater than Vit C which IC_50_ value is 3.1 µg/mL.

**Table 2:** Amounts of compound (μM) needed to inhibit acetylcholinesterase and antioxidant activities by 50%.

Compounds ^a^	DPPH ^b^	ABTS ^c^	TBARS ^d^	AChE ^e^
Isorhamnetin-7-*O*-β-D-glucopyranoside	1.73	21.76	7.20	55.70
Kaempferol	5.52	41.41	14.30	130.07
Quercetin-3,7-di-*O*-β-D-glucopyranosid	5.48	50.79	33.44	67.05
Quercetin	0.84	19.62	13.11	50.99
Quercetin-3′-*O*-β-D-glucoside	3.75	34.7	8.90	88.57
Isoquercetin	3.65	26.95	8.81	56.98
Hyperoside	11.19	113.25	7.47	94.61
Quercimeritin	3.69	24.91	6.85	52.3

^a^ Mean values of three replicates are given. Mean ± SD, n = 3; ^b^ Compared to Vit C (IC_50_ = 15.0 μM), IC_50_ for inhibition of DPPH radical formation; ^c^ Compared to Vit C (IC_50_ =17.6 μM), IC_50_ for inhibition of ABTS radical formation; ^d^ Compared to Vit C (IC_50_ = 29.5 μM), IC_50_ for inhibition of peroxidation of lipids, estimated as thiobarbituric acid reactive substances; ^e^ Compared to Huperzine A (IC_50_ = 0.042 μM), IC_50_ for inhibition of AChE.

Our data also showed that the free radical-scavenging activities of the compounds decrease with galactose, when compared to the corresponding glucose. The strongest scavenger was found to be quercetin, with IC_50_ equal to 19.62 μM in DPPH radical activity and 0.84 μM in ABTS radical activity. According to the molar concentration, it is obvious that the activity of quercetin is stronger than that of its glycosides. The overall evaluation of data also showed that antioxidant activities of those compounds and GHE on DPPH radical were lower than those of ABTS radical. All the compounds had good antioxidant activity compared with the positive control Vit C.

Lipid peroxidation involves the formation and propagation of lipid radicals with numerous deleterious effects, including destruction of membrane lipids, metabolic disorders and inflammation, and production of malondialdehyde (MDA) is a hallmark of this process [19]. Inhibition of lipid peroxidation was assessed by amount of MDA produced. Lipids in egg yolk undergo rapid nonenzymatic peroxidation in the presence of ferrous sulphate. GHE and the compounds showed good inhibition of lipid peroxidation. Phenolics present in plant sources have received considerable attention over the past decade because of their potential to prevent lipid peroxidation and diseases associated with it [19]. Therefore this suggests that the flavonoids in the GHE contributed significantly to the inhibition of lipid peroxidation.

For AChE inhibition, they showed weak activity. Among the flavonoids, the activity of quercetin and quercimeritin were shown to be higher than that of the other compounds and the IC_50_ values of AChE inhibition of these two compounds were equal to 50.99 and 52.3 µM, respectively. However, these compounds are shown to be nowhere near as effective as the well-known control AChE inhibitor huperzine A (0.042 μM).

### 2.3. Bioactives Provide Protection against Oxidative Stress in PC12 Cells

**Figure 3:**
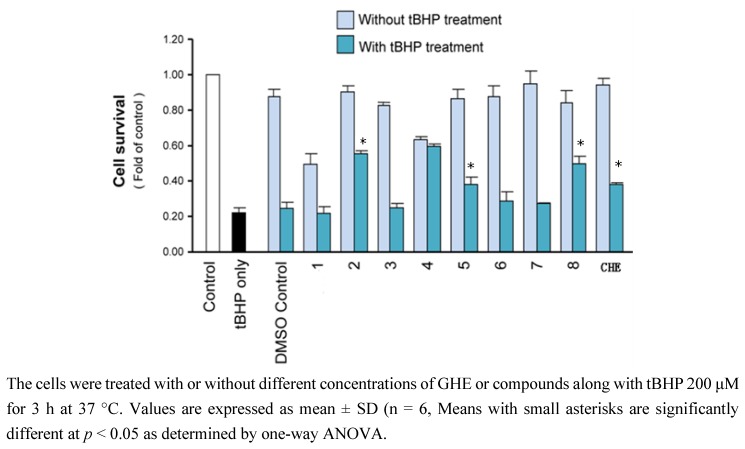
Protective effects of compounds and GHE on PC12 cells exposed to tBHP.

tBHP caused a reduction in cell viability with an IC_50_ value for PC12 cells death at 200 μM. PC12 cells were an established model of nerve growth factor (NGF) - induced neurite formation. Significant protection against tBHP-evoked cell death was demonstrated following pre-treatment with 100 μM compounds. In the control sample, the value of cell viability was set to be 100%. Under tBHP-induction oxidation, the cell viability was found to be reduced to ~20%. Cultures pre-treated with vitamin C have shown to protect the cells against oxidation significantly. For the drugs tested, it was shown that pre-treatment of several compounds, compound **2**, **5**, **8**, and GHE could exert the anti-oxidation effect in protecting the tBHP-induced cell death in PC12 cells (*p* < 0.05) (see Figure 3).

## 3. Experimental

### 3.1. Preparation of GHE and Isolation of Compounds

**Figure 4:**
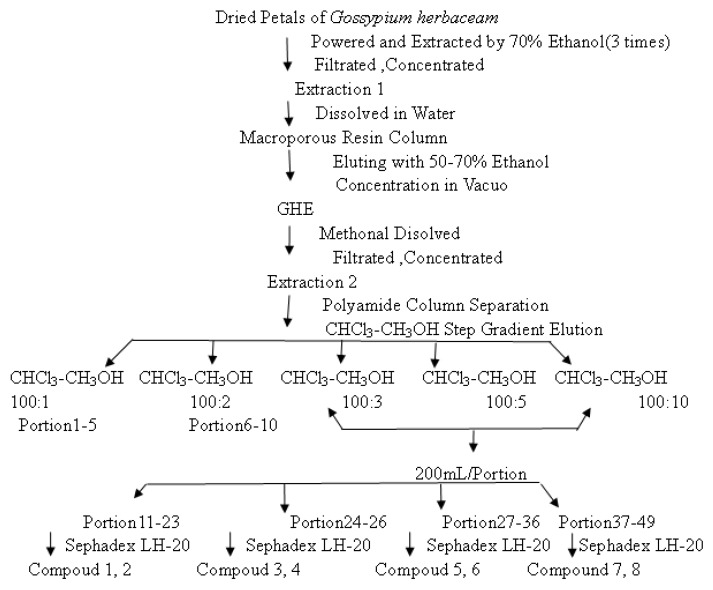
The extraction and fractionations of *Gossypium herbaceam*.

Flowers of *Gossypium herbaceam* were collected in the Xinjiang Uighur Autonomous Region, China. The dried petals were ground into a powder and extracted with 70% ethanol. After concentration under vacuum, the residue was dissolved in water, and subjected to macroporous resin column chromatography eluting with 50%–70% ethanol to give the fraction named GHE. Then GHE was chromatographed over a column of polyamide (15 g, 200–300 mesh) using a CHCl_3_-CH_3_OH step gradient elution, yielding 102 fractions. Fractions 11–23 were chromatographed on Sephadex LH-20 using CH_3_OH as eluent to yield compounds **1** (55 mg) and **2** (57 mg). Compound **3** (84 mg), **4** (446 mg), **5** (280 mg) and **6** (487 mg) were isolated from fractions 24–36. Compound **7** (26 mg) and **8** (208 mg) were isolated and purified from fractions 37–49 using Sephadex LH-20, separately (see Figure 4).

### 3.2. Chemicals

Acetylcholinesterase (AChE) type VI-S from electric eel, 5-5′-dithiobis-[2-nitrobenzoicacid] (DTNB), 1-1-diphenyl-2-picrylhydrazyl (DPPH), 2,2'-azinobis-(3-ethylbenzothiazoline-6-sulfonic acid) (ABTS), acetylthiocholine iodide (AChI), dimethyl sulfoxide (DMSO), ferrous sulfate (FeSO_4_·7H_2_O), thiobarbituric acid (TBA), trichloroacetic acid (TCA), potassium ferricyanide were obtained from Sigma (St. Louis, MO, USA). L-3-ketothreohexuronic acid lactone (vitamin C) and huperzine A were purchased from the National Institutes for Food and Drug Control, Beijing, China. All the other reagents were of analytical grade.

### 3.3. PC12 Cell Culture

Rat pheochromocytoma cells (PC12) cells were kindly donated by Professor Dong T. X. (The Hong Kong University of Science and Technology, Hong Kong, China). PC12 cells were maintained in 75 cm culture flasks containing 20 mL of RPMI-1640 media supplemented with 1% FBS, 1% L-glutamine, 1% non-essential amino acids and 1% penicillin/streptomycin. Cells were maintained in an incubator at 37 °C with 5% CO_2_. Experiments and cell maintenances were performed in a laminar flow hood under sterile conditions. Cells were passaged every 4–5 days and detached from flasks using 1× trypsin EDTA. Cell counts were performed using trypan blue to stain non viable cells, which were excluded from the count. Ninety-six well plates were used for cell viability experiments. The cells were then allowed to equilibrate for 24 h before the experiment.

### 3.4. Antioxidant Activity

#### 3.4.1. DPPH Radical Scavenging Assay

DPPH is a stable free radical that reacts with compounds that can donate a hydrogen atom. This method is based on the scavenging of DPPH through the addition of a radical species or an antioxidant that decolorizes the DPPH solution. The antioxidant activity is then measured by the extent of decrease in absorption at 515 nm. In brief, a 0.2 mM solution of DPPH in methanol was prepared, and 100 μL of this solution was added to 100 μL of the sample solution in methanol at varying concentrations. The solutions in the 96 well micro plates were shaken well and incubated in the dark for 30 minutes at room temperature. The absorbance was measured at 515 nm. A large decrease in the absorbance of the reaction mixture indicates substantial free radical scavenging activity of the compound. DPPH radical scavenging activity (%) = [(Abs_control_ − Abs_compound_)]/(Abs_control_)] × 100 where Abs_control_ is the absorbance of DPPH radical + methanol; Abs_compound_ is the absorbance of DPPH radical + compound/standard. The IC_50_ values (amount of compound required to decrease the absorbance of DPPH by 50%) were calculated using nonlinear regression analysis and vitamin C (Vit C) was for control.

#### 3.4.2. ABTS Radical Scavenging Assay

The ABTS radical scavenging method was developed by Rice-Evans and Miller in 1994 and was then modified by Rice-Evants *et al.* in 1999. The modification is based on the activation of metmyo-globin with hydrogen peroxide in the presence of ABTS^•+^ to produce a radical cation. This improved method generates a blue/green ABTS^•+^ chromophore via the reaction of ABTS and potassium persulfate and is now widely used. The antioxidant activity is then measured by the decrease in absorption at 734 nm. In brief, ABTS radical cation (ABTS^•+^) was produced by reacting ABTS stocking solution with 2.45 μM potassium persulfate and kept in the dark at room temperature for 12–16 h before use. The samples containing the ABTS^•+^ solution were diluted with methanol to obtain an absorbance of 0.700 (±0.02) at 734 nm and equilibrated at 30 °C. The ABTS^•+^ solution was freshly prepared for each assay. Different concentrations of compounds (16 μL) were allowed to react with 184 μL of the ABTS^•+^ solution and the absorbance was taken at 734 nm after 7 min using spectrophotometer. The ABTS^•+^ scavenging capacity of the compound percentage inhibition was calculated using formula: ABTS radical scavenging activity (%) = [(Abs_control_ − Abs_compound_)]/(Abs_control_)] × 100, where Abs_control_ is the absorbance of ABTS radical + methanol; Abs_compound_ is the absorbance of ABTS radical + compound/standard. The IC_50_ values (amount of compound required to decrease the absorbance of ABTS by 50%) were calculated using nonlinear regression analysis and Vit C was used as a control sample.

#### 3.4.3. Lipid Peroxidation Assay

A modified thiobarbituric acid-reactive species (TBARS) assay [20] was used to measure the lipid peroxide formed, using egg yolk homogenates as lipid-rich media. Adapted TBARS method was employed to measure the antioxidant capacity. Briefly, egg homogenate (100 μL of 1:25, v/v in phosphate-buffered saline (PBS) PH 7.4) and 10 μL of different concentrations of sample were added to a test tube as well as added 25 mmol/L freshly prepared FeSO4 made up to 300 μL with PBS. The homogenate was incubated at 37 °C for 15 min and then the reaction was stopped by adding 50 μL 15% w/v trichloroacetic acid (TCA) and centrifugation (3,500 rpm) for 15 min. An aliquot of 200 μL from supernatant was mixed with 100 μL thiobarbituric acid (TBA) and heated at 95 °C for 30 min. After cooling, absorbance of the samples was measured using a spectrophotometer at 532 nm.

### 3.5. Assay for Acetyl Cholinesterase (AChE) Inhibitory Activity

Inhibition of acetyl cholinesterase activity of samples was measured by the micro-plate assay adapted. AChE inhibitory activity was measured by slightly modifying the spectrophotometric method from the literature which was initially developed by Ellman [21]. Briefly, in this method, 100 μL of 0.1 mM sodium phosphate buffer (pH = 8.0), 20 μL of DTNB, 20 μL of test solution and 2 μL of AChE solution were added using a multichannel automatic pipette into a 96-well micro plate and incubated for 15 min at 25 °C. After incubation, acetylthiocholine iodide (100 μL of 0.05 mM water solution) was added as a substrate, and AChE activity was determined by UV spectrophotometry from the absorbance changes at 412 nm for 3.0 min at 25 °C. The concentration of the compounds which caused 50% inhibition of the AChE activity (IC_50_) was calculated via nonlinear regression analysis.

### 3.6. tBHP Exposure in PC12 Cells and Cell Viability Measurements

H_2_O_2_ is a well established pro-oxidant in PC12 cells over the concentration range used. tBHP shares some of the same properties of H_2_O_2_ but generates a more shallow neurotoxicity response over a wider concentration range than H_2_O_2_ in PC12 cells and elicits greater lipid peroxidation [22]. Cultured PC12 cells in 96-well-plate were pre-treated with the tested compounds derived from *Gossypium herbaceam* (100 μM for Compound **1** to **8**, and 100 µg/mL for GHE), vitamin C (100 μM; a positive control for anti-oxidation effect) for 24 h. For anti-oxidation effect, the cultures were treated with tBHP (200 μM) for 3 h. Cultures were subjected to cell viability test using the colorimetric MTT assay. Purple crystal inside the cells was dissolved by DMSO and measured by absorbance at 570 nm.

### 3.7. Statistical Analysis

All assays were undertaken in triplicate and all data were expressed as mean ± standard deviation for the number of experiments. One-way ANOVA was used to assess significant differences among the treatment groups. The concentration of tested samples required to inhibit 50% of the activity under the assay conditions was determined from dose-response curves and defined as the IC_50_ value.

## 4. Conclusions

All the compounds from GHE were identified as flavonoids. Flavonoids are one of the largest classes of plant secondary metabolites and are known to possess a number of important biological activities for human health [23]. A growing number of studies suggest that they have miscellaneous favorable biochemical and antioxidant effects potentially useful for the treatment of various diseases such as cancer, AD and atherosclerosis [24,25]. The antioxidant bioactivity of medicinal plants is partly attributable to their phenolic compounds, which constitute a diverse and ubiquitous group of phytochemicals in the plant kingdom. Flavonoids comprise the most common group of polyphenolic compounds and they exhibited potent antioxidant capacity when tested *in vitro *[26]. Flavonoids may also be capable of modulating glutamate excitotoxicity via scavenging of ROS [27]. Taken together, these observations suggest that the use of antioxidants may be useful in the treatment of AD.

Our previous study has demonstrated using a rat model of AD induced by Aβ that GHE possesses antioxidant stress properties [2] and found that GHE exhibited a beneficial inhibition on pro-apoptotic protein expression following ibotenic acid [12]. In this study, we showed that GHE exhibited certain activities against AChE and is an efficient free radical scavenger. A study of the bioactivities of these constituents was conducted to screen the activities to reveal the reason such as why GHE is effective, which may be helpful in preventing or alleviating patients suffering from AD as it showed both inhibitory activity of AChE and antioxidant activity. It may be important information for biologists who investigate this plant. Although the present experimental investigation showed GHE may provide some important clues to screening multipotent agents against AD, it is the first step towards a successful drug, much work is needed in the future to demonstrate the mechanism of action of GHE, as well as to identify the active components and their neuroprotective effects in models of AD.
